# Skeletal Muscle Fiber Size and Gene Expression in the Oldest-Old With Differing Degrees of Mobility

**DOI:** 10.3389/fphys.2019.00313

**Published:** 2019-03-26

**Authors:** Fabio Naro, Massimo Venturelli, Lucia Monaco, Luana Toniolo, Ettore Muti, Chiara Milanese, Jia Zhao, Russell S. Richardson, Federico Schena, Carlo Reggiani

**Affiliations:** ^1^Department of Anatomical, Histological, Forensic and Orthopedic Sciences, Sapienza University of Rome, Rome, Italy; ^2^Department of Neurosciences, Biomedicine and Movement Sciences, University of Verona, Verona, Italy; ^3^Department of Physiology and Pharmacology, Sapienza University of Rome, Rome, Italy; ^4^Department of Biomedical Sciences, University of Padova, Padua, Italy; ^5^Monsignor Arrigo Mazzali Foundation, Mantova, Italy; ^6^Division of Geriatrics, Department of Internal Medicine, The University of Utah, Salt Lake City, UT, United States; ^7^Department of Nutrition and Integrative Physiology, The University of Utah, Salt Lake City, UT, United States; ^8^Geriatric Research, Education, and Clinical Center, George E. Whalen VA Medical Center, Salt Lake City, UT, United States; ^9^Institute for Kinesiology Research, Science and Research Center of Koper, Koper, Slovenia

**Keywords:** aging, oldest-old, physical activity, muscle atrophy, single muscle fibers, myonuclei, gene expression

## Abstract

The oldest-old, in the ninth and tenth decades of their life, represent a population characterized by neuromuscular impairment, which often implies a loss of mobility and independence. As recently documented by us and others, muscle atrophy and weakness are accompanied by an unexpected preservation of the size and contractile function of skeletal muscle fibers. This suggests that, while most fibers are likely lost with their respective motoneurons, the surviving fibers are well preserved. Here, we investigated the mechanisms behind this fiber preservation and the relevance of physical activity, by comparing a group of 6 young healthy controls (YG: 22–28 years) with two groups of oldest-old (81–96 years), one able to walk (OW: *n* = 6, average 86 years) and one confined to a wheelchair (ONW *n* = 9, average 88 years). We confirmed previous results of fiber preservation and, additionally, observed a shift in fiber type, toward slow predominance in OW and fast predominance in ONW. Myonuclear density was increased in muscles of ONW, compared to YG and OW, potentially indicative of an ongoing atrophy process. We analyzed, by RT-qPCR, the expression of genes relevant for fiber size and type regulation in a biopsy sample from the vastus lateralis. In all oldest-old both myostatin and IGF-1 expression were attenuated compared to YG, however, in ONW two specific IGF-1 isoforms, IGF-1EA and MGF, demonstrated a further significant decrease compared to OW. Surprisingly, atrogenes (MURF1 and atrogin) expression was also significantly reduced compared to YG and this was accompanied by a close to statistically significantly attenuated marker of autophagy, LC3. Among the determinants of the metabolic fiber type, PGC1α was significantly reduced in both OW and ONW compared to YG, while AMPK was down-regulated only in ONW. We conclude that, in contrast to the shift of the balance in favor of pro-atrophy factors found by other studies in older adults (decreased IGF-1, increase of myostatin, increase of atrogenes), in the oldest-old the pro-atrophy factors also appear to be down-regulated, allowing a partial recovery of the proteostasis balance. Furthermore, the impact of muscle activity, as a consequence of lost or preserved walking ability, is limited.

## Introduction

Aging is accompanied by a decline in skeletal muscle mass and force with a significant impact on the everyday quality of life of the elderly (Janssen et al., 2002; Metter et al., 2002; Narici and Maffulli, 2010; Sayer et al., 2013). With advancing age, the architecture of skeletal muscle tissue is altered, with an increase of intramuscular adipose tissue, changes in the angle of pennation (Narici et al., 2003) and, moving down to cellular level, is associated with a reduction in muscle fiber cross sectional area, which becomes more pronounced when combined with disuse and inactivity (D’Antona et al., 2003; Suetta et al., 2007, 2009; Hvid et al., 2011). Of note, most of the available information on muscle aging comes from studies on individuals in the age range of 65–75, often identified as older-adult or young-old (Frontera et al., 1991, 2000; Larsson et al., 1997; Hughes et al., 2001; D’Antona et al., 2003; Ochala et al., 2007; Yu et al., 2007). However, in a recent study (Venturelli et al., 2015), members of our group examined not only much older subjects (∼90 years), usually identified as oldest-old, but also compared subjects characterized by different levels of mobility. The ninth decade of life is characterized by an accelerated decline of many functions and the loss of independent mobility is a common condition in non-agenarians. Indeed, according to epidemiological surveys, only approximately 30% of this segment of population is still able to walk (Christensen et al., 2008; Berlau et al., 2009). Our study design facilitated the isolation of the impact of disuse and advanced aging on muscle structure and function as we compared oldest-old subjects confined to a wheelchair with subjects still able to walk. Surprisingly, we found that the single fiber cross sectional area was preserved in both the oldest-old groups, while, in contrast, whole muscle volume and maximal voluntary force were dramatically reduced compared to young healthy subjects, particularly in the lower limbs of those who had lost the ability to walk. This observation was in partial agreement with the findings of Trappe and coworkers (Raue et al., 2009; Grosicki et al., 2016) who also studied a population of oldest-old individuals (∼85 years) and observed a preservation of skeletal muscle fiber size and a maintained or even improved capacity of single fibers to develop force during maximal calcium activation *in vitro*.

The conundrum of greatly diminished muscle size and function, while individual muscle fiber size and function are preserved, may potentially be explained by a loss of muscle fibers. In this respect, the neural system plays a pivotal role. Initially, with progressive motoneuron death and fiber denervation, and, then, by the disappearance of the denervated fibers or, possibly, by partial reinnervation of the surviving fibers by sprouting of slow motoneurons (Delbono, 2003, 2011; Payne and Delbono, 2004; Aagaard et al., 2010; Reid et al., 2012; Venturelli et al., 2018). Interestingly, it is still debated whether the loss of motoneurons can be slowed down by regular physical activity [see Power et al. (2010) in favor and Piasecki et al. (2016) against this view]. Unfortunately, the direct assessment of the impact of neural events on muscle fiber size and number during advanced age and disuse is somewhat complicated (Doherty et al., 1993). However, the comparison between the force developed during maximal voluntary contraction (MVC) and electrically stimulated contraction helps to estimate the contribution of reduced neural drive to muscle deconditioning (Venturelli et al., 2015). Furthermore, the evaluation of *in vivo* single twitch kinetics may further contribute to understand the functional condition of skeletal muscle, as the maximal rates of force development are clearly different among slow and fast motor units (Mero et al., 1991). Unfortunately, information regarding single twitch kinetics in the oldest-old is sparse.

Skeletal muscle fiber size is the result of a balance between protein synthesis and degradation and these two processes are regulated by very specific signaling pathways. Indeed, according to recent reviews of the literature in this field (Blaauw et al., 2013; Ciciliot et al., 2013), two major signaling pathways control protein synthesis, the IGF1–AKT– mTOR pathway, acting as a positive regulator, and the myostatin–Smad2/3 pathway, acting as a negative regulator. In turn, two major processes are responsible of protein degradation, the proteasomal and the autophagic–lysosomal pathways. The latter processes are controlled by a number of factors including FoxO transcriptional factors, atrogenes, and NF-kB (Schiaffino et al., 2013). However, the balance between these signaling pathways for the homeostatic control of skeletal muscle fiber size during advanced aging is not fully understood.

Therefore, this study sought to provide insight into the conundrum of whole muscle dysfunction and atrophy, but preservation of single fiber size by elucidating the expression of genes responsible for regulating skeletal muscle size. The analysis was carried out in a group of oldest-old people still able to walk and in a group confined to a wheelchair for at least 2 years, to assess the relevance of a preserved motor activity. We tested the hypothesis that, independent of the progressive neural system impairment associated with both advanced age and disuse, the balance between signals supporting protein synthesis and stimulating protein degradation is partially maintained. This balance results in the preservation of fiber size in the surviving muscle fibers, despite the marked overall loss of muscle fibers, the large reduction in whole muscle mass, and the decline in muscle force.

## Materials and Methods

### Participants

Eight young subjects (YG), 22–28 years old, and 15 oldest-old people, 81–96 years old, were enrolled in this study. The general characteristics of subjects are reported in Table 1. The oldest-old participants were approved to partake in this study based upon a physician’s assessment of minimal cognitive, cardiovascular, and musculoskeletal disease. This screening included, a health history, a physical examination, an evaluation of balance during sitting and ambulation (Tinetti, 1986), a blood pressure assessment, blood analyses, and a familiarization with the study procedures. The Ethics Committee of the Department of Neuroscience, Biomedicine and Motor Science of the University of Verona approved the study, and all experimental procedures were performed in accordance with the Declaration of Helsinki. Written, informed consent was obtained from all participants before inclusion in the study. The young subjects were normally physically active college students. The oldest-old participants were classified according to their ambulation capacity (Tinetti, 1986). Specifically, community dwelling subjects, able to walk independently (81–96 years old; *n* = 6) are identified here as OW, while, the oldest-old subjects with severe mobility limitation (81–92 years old; *n* = 9) are identified as ONW. The subjects were recruited among residents of the Monsignor Arrigo Mazzali Foundation, Geriatric Institute of Mantua, Italy. The medical staff recruited the oldest-old subjects that, at the time of the study, were mobility limited in the lower limbs (at least 2 years of being wheelchair bound) and unable to stand-up from a chair and walk independently (Tinetti, 1986). The diagnosis of altered gait capacity, poor balance, and an elevated risk of falling were the cause of this mobility restriction. Thus, three groups were formed and comparisons were carried out for each parameter between the three groups (see statistical analysis).

**Table 1:** Subject characteristics.

	YG = 8	OW = 6	ONW = 9
Age (years)	24 ± 3	86 ± 4 ^∗^	88 ± 5 ^∗^
Sex (F/M)	5/3	4/2	6/3
POMA Balance test (0–16)	16 ± 0	16 ± 0	3 ± 1
POMA Gait test (0–12)	12 ± 0	12 ± 0	0 ± 0
Body mass (kg)	61 ± 13	57 ± 12	57 ± 12
Body height (m)	1.66 ± 0.08	1.63 ± 0.07	1.65 ± 0.09
BMI (kg m^−2^)	22 ± 3	21 ± 4	21 ± 3
Body fat (%)	23 ± 3	32 ± 4 ^∗^	39 ± 5 ^∗†^
Sarcopenia index (kg m^−2^)	7.5 ± 2.3	5.4 ± 1.7	5.1 ± 0.6
Thigh muscle mass (kg)	5.3 ± 0.3	4.3 ± 0.3 ^∗^	3.3 ± 0.4 ^∗†^
Glucose (mg dl^−1^)	85 ± 4	96 ± 7 ^∗^	95 ± 6 ^∗^
RBC (10^6^ μl^−1^)	4.2 ± 0.1	4.1 ± 0.3	4.0 ± 0.3
Hb (g dl^−1^)	13.5 ± 0.5	11.5 ± 0.4 ^∗^	11.3 ± 0.7 ^∗^
HDL (mg dl^−1^)	50 ± 9	51 ± 8	53 ± 7
LDL (mg dl^−1^)	95 ± 11	106 ± 10 ^∗^	111 ± 13 ^∗^
SBP (mmHg)	119 ± 5	134 ± 6 ^∗^	137 ± 5 ^∗^
DBP (mmHg)	81 ± 5	84 ± 5	88 ± 9
Cognitive function MMSE (0–30)	–	27 ± 3	25 ± 3
**Comorbidity N (%)**			
Cardiovascular diseases	–	2 (33)	1 (11)
Diabetes	–	2 (33)	2 (22)
COPD	–	1 (17)	1 (11)
**Pharmacological treatments N (%)**			
Anti-depressive agents (Trazodone)	–	2 (33)	3 (33)
Oral anti-diabetics (Thiazolidinedione)	–	1 (17)	2 (22)

*YG, Young; OW, walking oldest-old, ONW, non-walking oldest-old; F, female; M, male; POMA, Performance-oriented assessment of mobility problems in elderly patients (Tinetti, 1986); BMI, body mass index, Sarcopenia index (Newman et al., 2003); RBC, red blood cells; Hb, hemoglobin; HDL, high-density lipoprotein; LDL, low-density lipoprotein; SBP, systolic blood pressure; DBP, diastolic blood pressure; MMSE, mini-mental state examination (Folstein et al., 1975); COPD chronic obstruction pulmonary disease. ^∗^Significantly different from YG; ^†^Significantly different from OW.*

### Cognitive Function

The *Mini Mental State Examination (MMSE)* was used to assess the global cognitive function of the participants (Folstein et al., 1975).

### Body Composition

Body fat and lean mass were assessed by means of DXA using a total body scanner (QDR Explorer W, Hologic, MA, United States; fan-bean technology, software for Windows XP version 12.6.1) according to the manufacturer’s procedures. The scanner was calibrated daily against the standard supplied by the manufacturer to avoid possible baseline drift. Whole body scanning took approximately 7 min. Data were analyzed using standard body region markers: upper and lower extremities, head, and trunk (pelvic triangle plus chest or abdomen). Additionally, the DXA scans were examined using non-standard body region markers to define thigh segments. The thigh region was delineated by an upper border formed by an oblique line passing through the femoral neck to the horizontal line passing through the knee (Skalsky et al., 2009). In agreement with the European consensus on definition and diagnosis of sarcopenia (Cruz-Jentoft et al., 2018), the level of sarcopenia was calculated as appendicular lean mass (aLM) corresponded to the sum of lean mass in the arms and legs, relative to height squared (Newman et al., 2003). All scanning and analyses were performed by the same operator to ensure consistency. In our laboratory the precision error (percent coefficient of variation with repositioning) of whole-body DXA measurements is 2.3, 0.5, and 2.8% for fat mass, lean mass, and percent fat mass, respectively.

### Electromyography

M-waves were recorded during femoral nerve stimulation in the vastus lateralis and biceps femoris muscles (detailed in next section). Pairs of full-surface solid adhesive hydrogel electrodes (H59P, Tyco Healthcare Group, Mansfield, MA, United States) were positioned lengthwise over the muscle belly, with an inter-electrode distance (center-to-center) of 20 mm. The ground electrodes were fixed over the ipsilateral patella. Light skin abrasion followed by skin cleansing kept electrical impedance below 10 kΩ. EMG signals were amplified with a pass-band of 10 Hz–1 kHz and digitized online at a sampling frequency of 5 kHz.

### Nerve Stimulation, Twitch Force and Kinetics

To record electrically evoked contractile responses of the quadriceps muscle, subjects were seated in an upright position with back support, in a custom made ergometer, as previously described (Venturelli et al., 2015). The hip and the knee were flexed at 90°, with the right ankle attached, via a strap and rigid steel bar, to a force transducer (DBBSE-100 kg, A2829. Applied Measurements Limited, Aldermaston, Berkshire, United Kingdom). The output from the force transducer was amplified (INT2-L, London Electronics Limited, Sandy Bedfordshire, United Kingdom), and recorded at a sampling rate of 5 kHz with a PowerLab-16/35 data acquisition system (ADInstruments, Bella Vista, NSW, Australia). Each test procedure began with the determination of the maximal M-wave and force responses. Briefly, current intensity was progressively increased from 0 mA to the value beyond which there was no further increment in M-wave amplitude. The stimulus utilized for the study was set at the 125% of the intensity required to produce a maximal M-wave response. To record the twitch response of the quadriceps muscle, the femoral nerve was stimulated with the cathode positioned in the femoral triangle, 3–5 cm below the inguinal ligament, and the anode placed over the iliac crest. The resting twitches (RT) were evoked in the passive muscle using electrical stimulation consisting of single square-wave pulses of 0.1 ms duration, delivered by a Digitimer DS7 constant-current stimulator (Digitimer Ltd., Welwyn Garden City, United Kingdom). Twitch peak amplitude, time to peak, maximal rate of force development between 10 and 90% of twitch peak (MRFD), maximal relaxation rate between 90 and 10% of twitch peak (MRFR), MFRD and MRFR relative to twitch peak (MRFD/F; MRFR/F), were determined for all RT (Sandiford et al., 2005; Maffiuletti et al., 2016).

### Skeletal Muscle Sampling

Skeletal muscle samples were obtained with a tru-cut needle (Paoli et al., 2010) from the vastus lateralis. Muscle samples were divided in three parts, the first immediately frozen in liquid nitrogen and used for molecular analysis, the second embedded in O.C.T. compound (Tissue-Tek), frozen in liquid nitrogen-cooled isopentane and used for morphological analysis, and the third immersed in skinning solution with 50% glycerol for single fiber dissection and myonuclei analysis. The samples were then stored at –80°C or at –20°C, if immersed in skinning solution (see below), with glycerol.

### Morphometry

Muscle samples, embedded in O.C.T., were cut into 10 μm thick cryosections with a cryostat (Thermo Fisher Scientific^TM^ CryoStar^TM^) maintained at –20°C and mounted on glass slides. Muscle sections were air-dried at room temperature rinsed 3 times for 5 min in 10% phosphate-buffered saline (PBS) and incubated for 60 min in goat serum at room temperature. Sections were then incubated for 1 h at room temperature with the primary antibody BA-F8 [1:50] against MHC type I. Following incubation with the primary antibody, the sections were rinsed 3 times for 5 min with PBS buffer and incubated for 1 h at room temperature with secondary antibody A21140 Alexa Fluor 350 goat anti mouse [1:800]. Then, the sections were rinsed 3 times for 5 min with PBS. Subsequently, the sections were incubated for 1 h at room temperature with primary antibodies SC-71 [1:200] against MHC type II, and ab11575 [1:200] against laminin. Following incubation with the primary antibodies, the sections were rinsed 3 times for 5 min with PBS buffer and incubated for 1 h at room temperature with secondary antibodies A21121 Alexa Fluor 488 goat anti mouse [1:800] and Ab96884 DyLight 550 goat anti rabbit [1:800]. Successively, the sections were rinsed 3 times for 5 min with PBS buffer and then dH_2_O.

BA-F8 and SC-71 antibodies were purchased from Development Studies Hybridoma Bank. Anti-laminin antibodies were purchased from the Abcam company. Secondary antibodies were purchased from Abcam and Invitrogen. Slides were visualized with an Axio Observer Z1 microscope (Carl Zeiss) using conventional wide field fluorescence microscopy as well as optical sectioning via structured-illumination fluorescence microscopy (Apotome, Carl Zeiss). The microscope was equipped with green (Excitation: BP 470/40 nm; Emission BP 525/50 nm) and blue (Excitation: BP 365/12 nm; Emission LP 397 nm) filters, an AxioCam HRm camera, and AxioVision software (Carl Zeiss). For fiber type distribution, all muscle fibers, on the cross section, were analyzed and a mean of 277 fibers were counted for each subject. Selected areas, corresponding to a mean of 91 fibers, were photographed and used for determination of fiber area using the public domain image–processing software, Image-J v1.46r (National Institute of Health, Bethesda, MD, United States).

### Myonuclei Counting

Single muscle fibers were manually dissected from the fiber bundles, immersed in skinning solution (mM composition: K propionate 150, Mg acetate 5, Na ATP 5, EGTA 5 and KH2PO4 5; relaxing solution KCl 100, imidazole 20, MgCl2 5, Na ATP 5 and EGTA 5), and fixed with 4% paraformaldehyde in PBS for 20 min at room temperature. After a short permeabilization with 0.1% Triton X-100 in PBS at room temperature, the fibers were incubated in 10% normal goat serum for at least 30 min to block non-specific antibody binding. Staining of Z lines with an anti α-actinin antibody was carried out to visualize the fiber segment and measure its size. Mouse anti α-actinin (clone EA-53 Sigma) was applied (1:2000) at room temperature in PBS, followed after 3 washes (10 min each), by fluorescent secondary Alexa-568 anti-mouse, (Molecular Probes) for 2 h at room temperature. To visualize nuclei, single fibers were stained with Hoechst (25 μg/ml; SIGMA) for 10 min. After a final wash in 0.1 M PB, the fibers were mounted in 100% glycerol (Sigma-Aldrich) and covered with a coverslip. The fibers were viewed with a confocal microscope (VICO, Nikon). Serial confocal optical sections (step size: 0.5 μm) were collected by scanning the fiber on the *z* axis from top to bottom. The fiber segment volume was reconstructed by adding the volume of the individual sections, each obtained as the product of thickness section (*z* axis) by surface area (*xy* axis). The sections were then collapsed on the *z* axis and the number of nuclei was counted. From nuclei number and fiber segment volume, the nuclear density (nuclei/10^6^ μm^3^) and the nuclear domain size (μm^3^/nucleus) were obtained. From fiber segment length and nuclei number, the longitudinal density (nuclei/mm) of the nuclei was determined.

### RNA Preparation and Analysis

High-quality RNA from muscle tissues was isolated using the RiboPure RNA Purification Kit (Ambion, Life Technologies, United States) according to the manufacturer’s instructions. The cDNA was prepared using the High Capacity cDNA Reverse Transcription kit (Applied Biosystems, Life Technologies, United States); the quantitative real-time PCR (RT-qPCR) was performed using the SYBR Green PCR Master mix (Applied Biosystems, Life Technologies, United States) according to the protocol for use in the Applied Biosystems 7500 Real-Time PCR System. For the quantification analysis, the comparative threshold cycle (Ct) method was used. The Ct values of each gene were normalized to the Ct value of cyclophilin in the same RNA sample. The gene expression levels were evaluated by fold change using the equation 2^−ddCt^. The primers used are reported in Table 2.

**Table 2:** Primers used in RNA analysis.

AKT1	Forward: 5′-CTGGTGCATCAGAGGCTGT-3′ Reverse: 5′-TTGATGTACTCCCCTCGTTTG-3′
AKT2	Forward: 5′-ACACAAGGAAAGGGAACCAG-3′ Reverse: 5′-ACCTAGCTCGGGACAGCTC-3′
AMPKα2	Forward: 5′-TACATTCTGGGTGACACGCT-3′ Reverse: 5′-TCCTACCACATCAAGGCTCC-3′
Atrogin	Forward: 5′-GCAGAGGCTGAGCGACGGG-3′ Reverse: 5′-GTTTGCGGATCTGCCGCTCG-3′
CyclophilinA or PPIA	Forward: 5′-TGTTCTTCGACATTGCCGT-3′ Reverse: 5′-TCTGTGAAAGCAGGAACCCT-3′
GDF-11	Forward: 5′-CTGGAGGAGGACGAGTACCA-3′ Reverse: 5′-GAACATCACCTTGGGGCTGA-3′
FOXO3A	Forward: 5′-CTACGAGTGGATGGTGCGTT-3′ Reverse: 5′-TCTTGCCAGTTCCCTCATTC-3′
IGF-1	Forward: 5′-GCTCTTCAGTTCGTGTGTGG-3′ Reverse: 5′-CGCAATACATCTCCAGCCTC-3′
IGF-1EA	Forward: 5′-GACATGCCCAAGACCCAGAAGGA-3′ Reverse: 5′-CGGTGGCATGTCACTCTTCACTC-3′
LC3 or MAP1LC3A	Forward: 5′-GCGACCAGCACCCCAGCAAA-3′ Reverse: 5′-GCGGCGCCGGATGATCTTGA-3′
IGF-1EC/MGF	Forward: 5′-CGAAGTCTCAGAGAAGGAAAGG-3′ Reverse: 5′-ACAGGTAACTCGTGCAGAGC-3′
mTOR	Forward: 5′-CATTGTTCTGCTGGGTGAGA-3′ Reverse: 5′-TCCGGCTGCTGTAGCTTATT-3′
MURF-1 or TRIM63	Forward: 5′-ACGAGGTGATCATGGATCGT-3′ Reverse: 5′-CTTCGTGCTCCTTGCACAT-3′
Myostatin or GDF-8	Forward: 5′-TGTAACCTTCCCAGGACCAG-3′ Reverse: 5′-AGAGGGTAACGACAGCATCG-3′
PGC-1α	Forward: 5′-GGTGCAGTGACCAATCAGAA-3′ Reverse: 5′-AATCCGTCTTCATCCACAGG-3′
Sirt-1	Forward: 5′-GCTCGCCTTGCTGTAGACTT-3′ Reverse: 5′-TGTGACAGAGAGATGGCTGG-3′

### Statistical Analysis

The data collected in each group of subjects are presented as the mean ± standard deviation (s.d.) or standard error of the mean (s.e.m.), as indicated. Statistical significance of the difference between means was determined with repeated measures ANOVA followed by Bonferroni *post hoc* test. GraphPad Prism software was used for all analysis.

## Results

### Muscle and Fiber Atrophy

**FIGURE 1:**
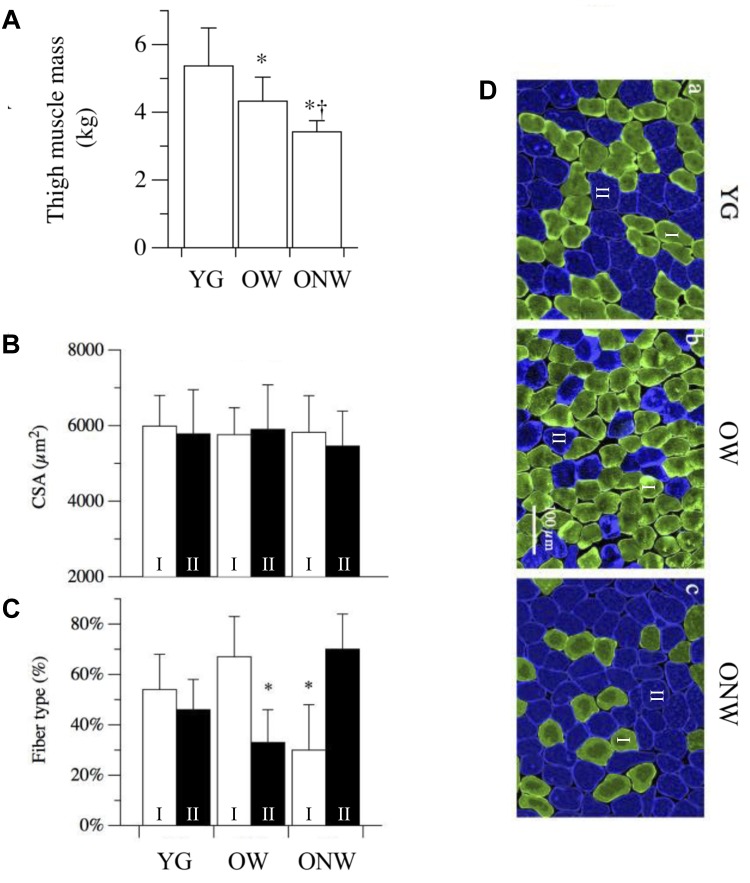
Variations in thigh muscle mass, fiber cross sectional area and fiber type distribution in vastus lateralis of young (YG), walking oldest-old (OW), and non-walking oldest-old (ONW). **(A)** Thigh muscle mass, determined with DEXA, documents the significantly lower values in OM and OI compared to YG and even lower values in ONW compared to OW (YG *n* = 8, OW *n* = 6, and ONW *n* = 9). **(B)** Single muscle fiber cross sectional area was not different between the three groups. **(C)** Muscle fiber types, determined with anti-myosin antibodies, documents a significant difference in OW and ONW compared to YG. **(D)** Examples of sections of biopsy samples stained with anti-slow myosin antibody (green) and anti-fast myosin antibody (blue). Data in panels **A–C** are presented as mean ± S.E.; ^∗^Significantly different from YG subjects; ^†^Significantly different from OW subjects.

To characterize the degree of muscle atrophy we determined thigh muscle mass and muscle fiber cross sectional area in the three groups of subjects (YG, OW, ONW). Overall both OW and ONW participants were classified as sarcopenic according to the cut-points of 7.23 kg m^−2^ (men) and 5.67 kg m^−2^ (women) (Table 1). As documented in Figure 1A, the thigh muscle mass, determined with DXA, revealed significantly lower values in the old compared to young (*p* < 0.05) and even lower values in ONW compared to OW (*p* < 0.05). In contrast, the determination of single muscle fibers cross sectional area did not reveal significant differences among the average values in the three groups (Figure 1B). The counting of the fibers, identified as slow or fast with specific anti-myosin antibodies, revealed that, in the vastus lateralis, the proportion of slow fibers was increased in OW (*p* < 0.05) and decreased in ONW in comparison to YG (*p* < 0.05) (Figure 1C,D).

To assess the impact of aging on muscle fibers, we analyzed the myonuclear density and the volume of the myonuclear domain in single muscle fibers isolated from the biopsy samples of vastus lateralis of the three groups of subjects. The results, reported in Figure 3, indicate distinct variations in the fibers of the oldest-old subjects in relation to their ability to walk. When compared to young, fibers from ONW subjects exhibited a significant increase (*p* < 0.05) in myonuclei number per unit of length and per unit of volume, while the corresponding reduction in nuclear domain size did not reach statistical significance. Minor and not significant variations in the opposite directions were observed in OW compared to YG.

**FIGURE 2:**
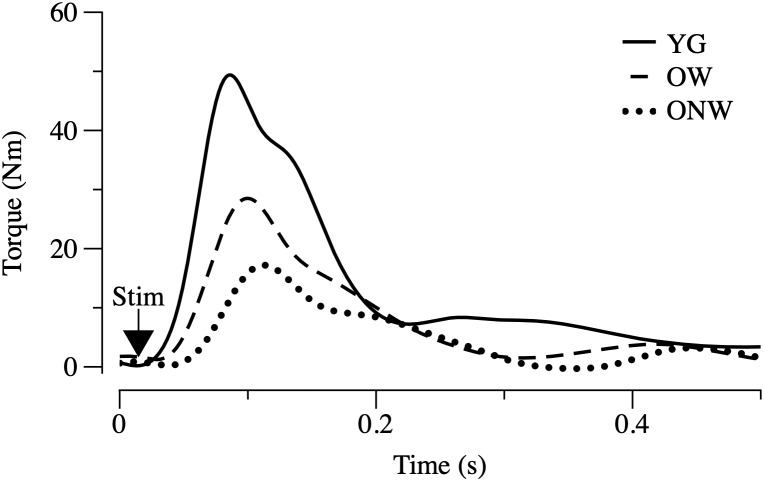
Electrically evoked twitch in young (YG), walking oldest-old (OW), and non-walking oldest-old (ONW). Examples of twitch recorded in leg extensor muscles after single pulse stimulation are illustrated. Protocol of stimulation and torque recording are described in Section “Materials and Methods.” Values of the time and torque parameters are reported for the three groups in Table 3.

The functional characterization of the leg extensor muscles was based on the determination of twitch response induced by electrical stimulation, as illustrated in Figure 2 and reported in Table 3. The force parameters (twitch peak, MRFD and MRFR) were reduced in OW and ONW in direct relation to the reduced muscle mass. The rates of force development and relaxation (MRFD/peak and MRFR/peak) were significantly reduced in both OW and ONW compared to YG subjects (*p* < 0.05). Further significant prolongation of the time to peak was detectable in ONW compared to OW (*p* < 0.05).

**FIGURE 3:**
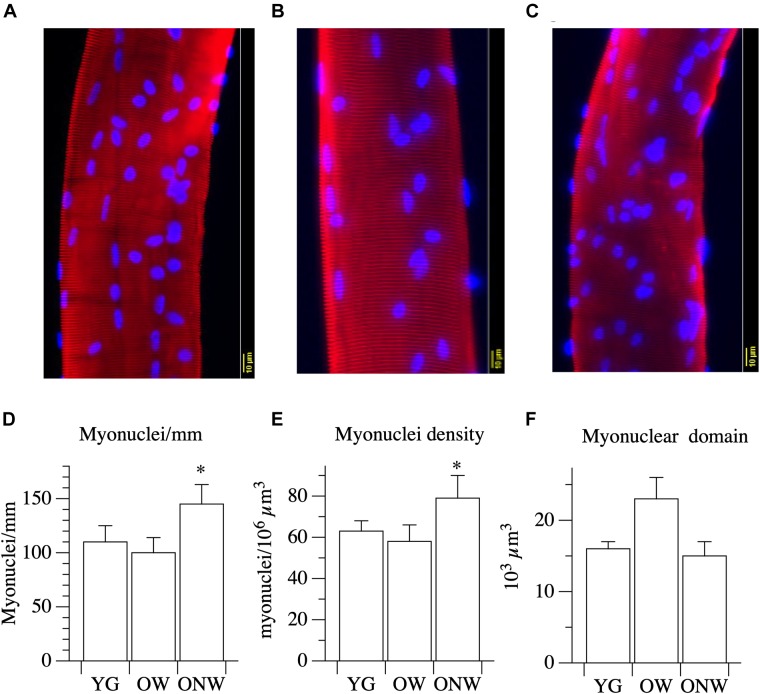
Myonuclear density and myonuclear domain size determined in single muscle fibers from young (YG), walking oldest-old (OW), and non-walking oldest-old (ONW). **(A–C)** typical examples of segment of single fibers stained for nuclear visualization: YG **(A)**, OW **(B)** and ONW **(C)**, **(D)** longitudinal density expressed as number of nuclei per mm of fiber, **(E)** nuclear density, expressed as number of myonuclei per million of cubic micrometers, **(F)** nuclear domain size expressed in cubic micrometers. Data are presented as mean ± S.E.; ^∗^Significantly different from YG.

**Table 3:** Parameters of the electrical evoked twitch response.

	Twitch peak Nm	Time to peak ms	MRFD Nm s^−1^	MRFR Nm s^−1^	MRFD/peak s^−1^	MRFR/peak s^−1^
YG	46.1 ± 3.1	85 ± 5	879 ± 20	181 ± 8	19.2 ± 1.6	3.9 ± 0.3
OW	27.5 ± 4.0 ^∗^	94 ± 3^∗^	461 ± 66^∗^	70 ± 12^∗^	17.0 ± 3.2	2.6 ± 0.7^∗^
ONW	18.8 ± 3.98^∗^$	106 ± 5^∗^$	366 ± 37^∗^$	49 ± 9^∗^$	20.1 ± 4.2	2.7 ± 0.9^∗^

*Twitch peak amplitude in Nm, time to peak in ms, maximal rate of force development between 10 and 90% of twitch peak (MRFD in Nm s^-1^), maximal relaxation rate between 90 and 10% of twitch peak (MRFR in Nm s^-1^), MFRD and MRFR relative to twitch peak (MRFD/peak; MRFR/peak). The latter two parameters are pure rates, with no relationship to force development. (YG n = 8, OW n = 6, and ONW n = 9). Data are presented as mean ± S.E; ^∗^Different from YG (p < 0.05); $ Different from OW (p < 0.05).*

### Quantitative Expression Analysis of Genes Affecting Muscle Growth

**FIGURE 4:**
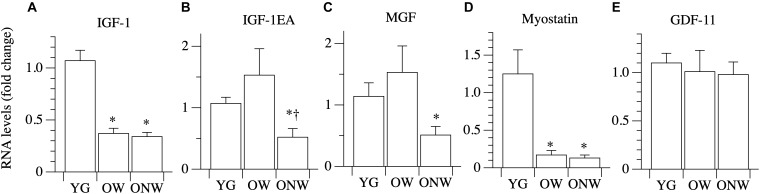
Real time PCR of genes coding for factors affecting muscle growth in young (YG), walking oldest-old (OW), and non-walking oldest-old (ONW). **(A)** IGF-1, **(B)** IGF-1EA, **(C)** MGF or IGF-1EC, **(D)** Myostatin, **(E)** GDF-11. The mRNA levels of the indicated genes were determined by RT-qPCR on muscle obtained by biopsies from YG, OW, and ONW. mRNA levels are expressed as fold change compared to the levels in the vastus lateralis samples of the young (YG *n* = 8; OW *n* = 6 ONW *n* = 9). Data are presented as mean ± S.E.; ^∗^ Significantly different from YG subjects; ^†^Significantly different from OM subjects.

To unravel the mechanisms behind the preservation of skeletal muscle fiber thickness in the elderly, we explored the mRNA expression levels of key genes involved in the control of protein synthesis and degradation by quantitative real-time PCR (Figure 4). IGF-1 mRNA total expression and the expression of the two IGF-1 isoforms (IGF-1EA, IGF-1EC or MGF) were determined in the vastus lateralis muscle of young and oldest-old subjects. The comparison between YG and both OW and ONW indicated a decreased expression of total IGF-1 in the oldest-old people (*p* < 0.01). Regarding the expression of muscle specific isoforms, IGF-1EA and MGF were almost unaffected in OW compared to YG. The comparison between OW and ONW indicated a clear and significant decrease in expression of both IGF-1EA and MGF in ONW. Next, we determined level of expression of myostatin (also called GDF-8). The myostatin mRNA levels were similarly reduced in the vastus lateralis muscle of both OW and ONW (*p* < 0.05). We also determined the level of expression of another member of TGF-β family, GDF-11, which has been related to the control of muscle mass. No variation of GDF-11 expression was found in both OW and ONW in comparison to YG (Figure 4).

**FIGURE 5:**
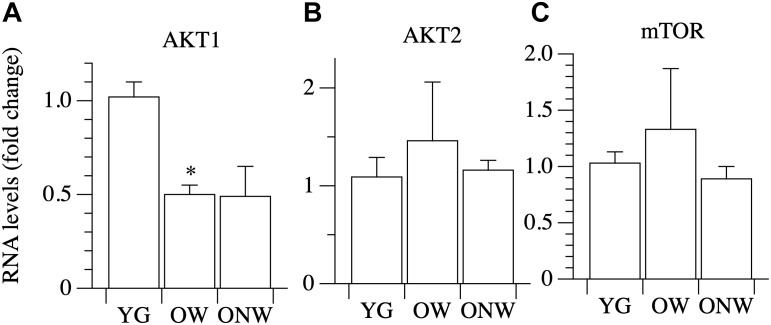
Real time PCR of genes coding for factors involved in protein synthesis in young (YG), walking oldest-old (OW), and non-walking oldest-old (ONW). **(A)** AKT1, **(B)** AKT2, **(C)** mTOR. The mRNA levels of the indicated genes were determined by RT-qPCR on muscle obtained by biopsies from YG, OW, and ONW. mRNA levels are expressed as fold change compared to the levels in the vastus lateralis samples of the young (YG *n* = 8; OW *n* = 6 ONW *n* = 9). Data are presented as mean ± S.E.; ^∗^Significantly different from YG subjects.

The intracellular signaling pathway which links IGF-1 binding to the receptor to an increased protein synthesis in the ribosome is based on three major kinases: phosphoinositide-3-kinase (PI3K), AKT/Protein Kinase B (Akt/PKB) and mammalian target of rapamycin (mTOR). We assessed the expression of AKT1, AKT2, and mTOR in vastus lateralis muscle, and we found a decrease in the level of AKT1 mRNA expression, which reached statistical significance only in the muscles of OW (*p* < 0.01) (Figure 5).

### Protein Degradation Pathways

**FIGURE 6:**
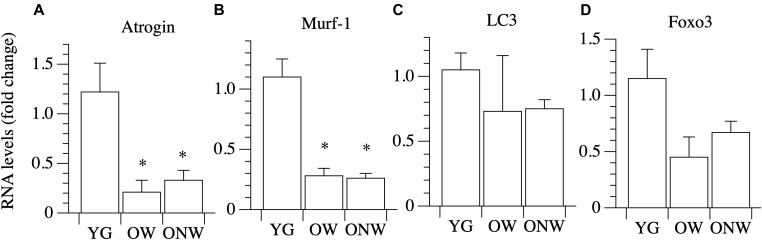
Real Time PCR of genes coding for factors involved in protein degradation in young (YG), walking oldest-old (OW), and non-walking oldest-old (ONW). **(A)** Atrogin, **(B)** Murf-1, **(C)** LC3, **(D)** Foxo3. The mRNA levels of the indicated genes were determined by RT-qPCR on muscle obtained by biopsies from YG, OW, and ONW. mRNA levels are expressed as fold change compared to the levels in the vastus lateralis samples of the young. (YG *n* = 8; OW *n* = 6; ONW *n* = 9). Data are presented as mean ± S.E.; ^∗^ Significantly different from YG subjects.

We analyzed genes coding for the muscle specific atrophy-related ubiquitin ligases Atrogin-1 and MuRF-1 (Figure 6A,B). The expression of both genes was down regulated in the vastus lateralis muscle of both OW (*p* < 0.05 and *p* < 0.01) and ONW (*p* < 0.01 for both genes) compared to the YG, but there was no modification in their expression between OW and ONW.

The expression of the autophagy-related gene LC3 was slightly, but not significantly, modulated in the vastus lateralis muscle of both OW and ONW compared to YG and with no significant difference between the two groups of the oldest-old (Figure 6C).

We then determined the expression of transcriptional factor Foxo3A and observed a trend toward values being lower in the OW and ONW than in the YG, although the variations did not reach statistical significance (Figure 6D).

### Energy Sensing Factors

**FIGURE 7:**
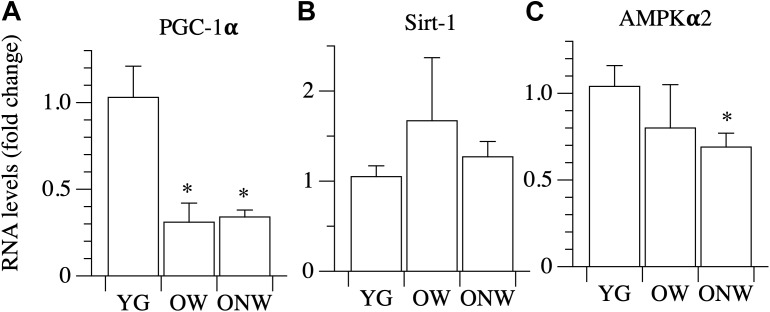
Real time PCR of genes coding for energy sensing factors in young (YG), walking oldest-old (OW), and non-walking oldest-old (ONW). **(A)** PGC-1α, **(B)** Sirt-1, **(C)** AMPKα2. The mRNA levels of the indicated genes were determined by RT-qPCR on muscle obtained by biopsies from YG, OW, and ONW. mRNA levels are expressed as fold change compared to the levels in the vastus lateralis samples of the young (YG *n* = 8; OW *n* = 6; ONW *n* = 9). Data are presented as mean ± S.E.; ^∗^Significantly different from YG subjects.

Among the factors involved in energy sensing and mitochondrial biogenesis regulation, we analyzed the gene expression of PGC-1α, the master gene controlling mitochondrial biogenesis, and two metabolic sensors, SIRT-1, AMPKα2, which act upstream of PGC-1α. No differences were found of SIRT-1 expression in the vastus lateralis muscle of the YG, OW, or the ONW (Figure 7). The level of AMPKα2 expression was determined to be significantly decreased only in ONW group (*p* < 0.05, Figure 7). The level of PGC-1α expression was determined to be decreased (*p* < 0.01) in the vastus lateralis muscle of oldest-old, both the OW and ONW in comparison to the YG (Figure 7).

## Discussion

The loss of independent mobility becomes a very common condition with advanced age. Thus, for many of the oldest-old (i.e., people 85 years of age or greater, see e.g., (Zizza et al., 2009), the effects of disuse combine or overlap with the impact of aging itself. Evidence published by our team (Venturelli et al., 2015) and others (Raue et al., 2009) documents a dissociation between muscle impairment and atrophy and single muscle fiber size and contractile properties in the oldest-old. In this study, we aimed to elucidate the expression of genes responsible for regulating skeletal muscle fiber size in the lower limbs of the oldest-old still able to walk (OW) and the oldest-old confined to a wheelchair (ONW), each in comparison with young, healthy, individuals (YG). As anticipated (Venturelli et al., 2015), muscle atrophy was evident in both of the oldest-old groups compared to YG, but was more pronounced in ONW than OW, whereas average fiber size was preserved in both oldest-old groups. The present results, however, revealed that, despite similar fiber size, the impact of the loss of the ability to walk was detectable in fiber type distribution, myonuclear density, and gene expression. Actually, the gene expression analysis documented that, in the muscle of the oldest-old, the expression of the positive regulator of protein synthesis IGF-1 was attenuated and so too was the negative regulator myostatin. Coupled with no change in some IGF-1 isoforms, IGF-IEA and IGF-1EC/MGF, and the attenuation of genes responsible for protein degradation (Atrogin, Murf-1, FoXO3 and LC3), the emerging picture is suggestive of a somewhat preserved balance between pro- and anti-atrophy factors.

The determination of thigh muscle mass confirmed the marked age-related skeletal muscle atrophy which was more pronounced in the subjects who were confined to a wheelchair. This loss of muscle mass was accompanied by an approximately proportional decrease in muscle strength, determined *in vivo* with a single electrically stimulated twitch. When the effect of advanced age on the muscle cells was assessed by measuring the cross-sectional area of single fibers, there was no significant difference between the average values obtained in YG and either of the oldest-old groups in agreement with previous findings by our group and others (Raue et al., 2009; Venturelli et al., 2015; Grosicki et al., 2016). This mismatch implies that, at least in the oldest-old, the age-related atrophy is due to the loss of muscle fibers rather than a decrease in fiber size. Here it is worth noting that the immunohistochemistry analysis revealed two very different fiber type distributions as the consequence of advanced aging (OW, predominantly type I or slow fibers) and advanced aging and disuse (ONW, predominantly type II or fast fibers). It should also be recognized that in the skeletal muscle of elderly subjects, the precise identification of fiber type is often uncertain as the proportion of hybrid slow-fast fibers increases significantly (Klitgaard et al., 1990; Rowan et al., 2012). Thus, employing simple fiber counting after immunohistochemical staining with anti-myosin antibodies, hybrid fibers will be considered either slow or fast, depending on the proportion of the myosin isoform expressed. However, the changes observed in the current study seem to provide clear evidence of a fiber type shift. An additional difference in the fibers of the subjects confined to the wheelchair compared to the subjects still able to walk and, this time, also compared to the young, was the increased nuclear density. A similar increase in myonuclei number and density with aging has previously been reported in murine (Brack et al., 2005) and human (Cristea et al., 2010) muscles. It is not clear whether this change is a cause or an effect of fiber atrophy with aging. However, as the number of myonuclei increases in proportion to fiber size during fiber growth, but does not decrease when fibers return the original size, the high nuclear density is suggestive of atrophy (Gundersen and Bruusgaard, 2008). This observation helps to explain why, despite exhibiting a predominance of type II fibers, which are generally larger than type I fibers, the type II fibers of the ONW actually tended to be smaller. However, to reiterate, despite these difference in muscle phenotype and muscle size, there was still no significant difference in the average fiber size between the YG and either of the oldest-old groups.

Although skeletal muscle is exposed to numerous factors that may affect skeletal muscle fiber size, including both circulating IGF-1, which has been documented to decrease with age (Perrini et al., 2010), and circulating myostatin, which has been determined to increase with age, at least in elderly women (Bergen et al., 2015), perhaps the most significant factors are expressed and released by the muscle fibers themselves. Specifically, fibers express pro-atrophic and pro-hypertrophic factors, which act in paracrine manner on the muscle. To investigate, at the molecular level, why, with advanced age, the whole muscle is atrophied, but single muscle fiber size is preserved, this study focused on the major paracrine-like factors involved in the regulation of muscle fiber size, including IGF-1, and its two isoforms IGF-1EA and IGF-1EC/MGF, myostatin, and GDF-11. The real time PCR analyses revealed that, compared to the YG, the OW exhibited significantly attenuated expression of total IGF-1 and myostatin, but there was no difference in the expression of IGF-1EA, MGF and GDF-11. These data are intriguing because they suggest that the age-dependent atrophy of the whole muscle is not immediately explained by a tipping of the balance between pro-atrophy and pro-hypertrophy signals, as the decrease in the expression of IGF-1 was balanced by a concomitant decrease in the expression of myostatin. The decrease of IGF-1 expression, reported here, is in agreement with previous observations from skeletal muscle biopsies in old people, which documented a 25–45% age-related decrease in IGF-1 mRNA gene expression (Welle et al., 2002; Leger et al., 2008). In contrast to the present findings, however, previous studies documented either elevated myostatin mRNA expression (Leger et al., 2008) or no change (Welle et al., 2002) as a consequence of aging. Indeed, this study appears to be the first to clearly document a decrease in myostatin in skeletal muscle with aging, in the oldest-old, who with an average age close to 90 years, are 15–20 years older than the subjects assessed in the majority of other aging studies. Furthermore, likely also counteracting the fall in both IGF-1 and myostatin, the expression of the two specific IGF-1 isoforms was not attenuated in OW. This suggests a transcriptional re-organization, which may also contribute to the maintenance of muscle fiber size. Of note, in the ONW, the oldest-old group with the most atrophied whole muscle due to the combination of both advanced age and inactivity, both IGF-1EA and MGF were significantly attenuated, but still average muscle fiber size was unchanged. Finally, the oldest-old did not exhibit changes in expression of GDF-11, which has been reported to increase with age in rat muscle and in human serum, an increase which has been related to muscle atrophy (Sinha et al., 2014; Egerman et al., 2015). Thus, unchanged GDF-11 gene expression coupled with a fall in both IGF-1 and myostatin, in addition to unchanged IGF-1 isoforms in the OW, may explain, at least in part, the preservation of muscle fiber size in the oldest-old.

We found that the level of expression of two important effectors of the IGF-1 signaling pathway, such as AKT2 and mTOR, are not modified by aging, whereas AKT1 expression is decreased. While AKT2 is mainly involved in glucose metabolism, AKT1 is part of the protein synthesis pathway. AKT regulation, however, is mainly evoked at the post-translational phosphorylative level. Furthermore, the direct target of AKT inhibitory action, Foxo3, is down regulated in the vastus lateralis of the oldest-old subjects as well as important genes controlled by Foxo3 and involved in muscle atrophy, such as Atrogin and MURF-1. These data suggest that the down-regulation of factors which trigger the degradation of the protein in muscle cells could play a pivotal role in maintaining the integrity of the skeletal muscle fiber in the oldest-old. Additionally, there was a trend for LC3 (also called MAP1LC3A) gene expression to decrease in vastus lateralis muscle of the oldest-old, although this did not achieve statistical significance. LC3 codes for a protein involved in autophagosome vesicle assembly and can be considered a marker of autophagy. It is worthy of note that, although directed to protein degradation, the mechanism of autophagy is essential to preserve muscle fiber size, as demonstrated by the genetic manipulation of the genes coding for the essential component of the autophagic vesicle (Masiero et al., 2009; Carnio et al., 2014; Jiao and Demontis, 2017).

The current results indicate that the average size of a single muscle fiber is not modified by aging or use, but the histotype of the fibers is significantly modified. In fact, the proportion of slow fibers in the vastus lateralis muscle of OW was increased compared to the corresponding proportion in the YG. In contrast, the number of slow fibers declined in the muscle of ONW. The increase in the number slow fibers in the OW could be either a consequence of a transition from fast-to-slow fibers as a consequence of aging or, as an alternative, may be the result of a specific loss of type II, fast glycolytic, fibers which are most vulnerable to the effects of aging in contrast to the more resistant type I slow oxidative fibers (Anderson and Prolla, 2009). Following a similar line of reasoning, the increase of fast fibers in ONW could be the result of disuse which can cause a slow-to-fast fiber transition [see for a review Blaauw et al. (2013)]. To address this issue, we analyzed the expression of two factors which are implicated in the fast-to-slow and glycolytic-oxidative transitions: SIRT1 and PGC1-α (Chalkiadaki et al., 2014). The mRNA level of SIRT1, a protein deacetylase, correlated with the fiber type transition as it was slightly, but not significantly, increased in OW oldest-old subjects compared to YG. In contrast, the mRNA level of PGC1-α, a protein involved in mitochondrial biogenesis, was dramatically reduced in both groups of oldest-old subjects as already reported in previous aging studies in humans and murine models (Chan and Arany, 2014). Interestingly, PGC1-α levels were not modified by differences in muscle activity for locomotion, suggesting that PGC1-α expression is more connected with the status of the aged skeletal muscle than with a fast-to-slow fiber transition. Therefore, on the whole, these findings support the hypothesis that the decrease of muscle mass observed with aging is due to a loss of fast fibers rather than an increase of the percentage of slow fibers. The combined impact of disuse and aging, however, might lead to a slow-to-fast fiber transition, as occurs in other conditions of muscle disuse and unloading (Schiaffino and Reggiani, 2011; Blaauw et al., 2013).

AMP-activated protein kinase (AMPK) is a key energy-sensitive enzyme that controls numerous metabolic and cellular processes. Previous studies showed a decrease in AMPKα2 activity in vastus lateralis muscle and lower levels of AMPK phosphorylation in the elderly (Li et al., 2012). In the present study, there was evidence of a slight decrease of AMPKα2 mRNA expression in the vastus lateralis of the oldest-old, more evident and statistically significant in ONW than in OW. This observation is in agreement with the finding, in a previous study, that AMPKγ3 subunit protein content was lower in the immobilized leg than in the contralateral leg of older men (Vigelso et al., 2016), suggesting an impaired glucose utilization with aging, which is exacerbated by the lack of movement (Vigelso et al., 2016).

In conclusion, this study confirmed the preservation of the average muscle fiber size in the oldest-old, whether able to walk or confined to a wheelchair, and provided initial insight into the cellular signal network involved in this preservation. In contrast to the commonly documented tipping of the balance in favor of pro-atrophy factors in older adults (decrease in IGF-1 and increases in both myostatin and atrogenes), in the oldest-old the pro-atrophy factors appear to be down-regulated, allowing a partial recovery of the proteostasis balance. Interestingly, the impact of a preserved capacity for locomotion is rather limited, detectable in the fiber type distribution and in the expression of few genes, but with no effect on single fiber cross sectional area. It remains to be established whether the preservation of single fiber cross sectional area is a compensatory response to the age-related loss of motor units and muscle fibers or whether this is a specific response from a sub-population of fibers which are not only able to survive the widespread motoneuron and fiber death, but also to preserve their trophic features. Additionally, the failure of the muscle fibers of the oldest-old to increase in thickness with resistance training (Raue et al., 2009) and the lack of significant difference between walking and not-walking subjects observed in this study, support the view that, in this specific group of old people, the preservation of fibers size is a consequence of a selective process and not of the adaptative response to the progressive loss of motor units. This conclusion implies that the benefit of physical activity in this extreme population is limited, at least at the muscle fiber level.

## Author Contributions

FN, MV, RR, FS, and CR conceived and designed the study. FN, MV, LM, LT, EM, JZ, RR, FS, and CR performed the experiments. FN, MV, LM, LT, EM, JZ, CM, and CR analyzed the data. FN, MV, LT, RR, CM, and CR interpreted the results. MV, LT, JZ, and CR prepared the figures. FN, MV, RR, FS, and CR drafted the manuscript. MV, RR, and CR edited the manuscript.

## Conflict of Interest Statement

The authors declare that the research was conducted in the absence of any commercial or financial relationships that could be construed as a potential conflict of interest.
