# Corrigendum: Skeletal Muscle Fiber Size and Gene Expression in the Oldest-Old With Differing Degrees of Mobility

**DOI:** 10.3389/fphys.2020.00127

**Published:** 2020-02-25

**Authors:** Fabio Naro, Massimo Venturelli, Lucia Monaco, Luana Toniolo, Ettore Muti, Chiara Milanese, Jia Zhao, Russell S. Richardson, Federico Schena, Carlo Reggiani

**Affiliations:** ^1^Department of Anatomical, Histological, Forensic and Orthopedic Sciences, Sapienza University of Rome, Rome, Italy; ^2^Department of Neurosciences, Biomedicine and Movement Sciences, University of Verona, Verona, Italy; ^3^Department of Physiology and Pharmacology, Sapienza University of Rome, Rome, Italy; ^4^Department of Biomedical Sciences, University of Padova, Padua, Italy; ^5^Monsignor Arrigo Mazzali Foundation, Mantova, Italy; ^6^Division of Geriatrics, Department of Internal Medicine, The University of Utah, Salt Lake City, UT, United States; ^7^Department of Nutrition and Integrative Physiology, The University of Utah, Salt Lake City, UT, United States; ^8^Geriatric Research, Education, and Clinical Center, George E. Whalen VA Medical Center, Salt Lake City, UT, United States; ^9^Institute for Kinesiology Research, Science and Research Center of Koper, Koper, Slovenia

**Keywords:** aging, oldest-old, physical activity, muscle atrophy, single muscle fibers, myonuclei, gene expression

In the original article, there was an error in citing a paper concerning the impact of physical activity on motoneuron survival: Dranseika et al. (2016) was cited instead of Piasecki et al. (2016). A correction has been made to second paragraph of the Introduction, which reads as follows:

The conundrum of greatly diminished muscle size and function, while individual muscle fiber size and function are preserved, may potentially be explained by a loss of muscle fibers. In this respect, the neural system plays a pivotal role. Initially, with progressive motoneuron death and fiber denervation, and, then, by the disappearance of the denervated fibers or, possibly, by partial reinnervation of the surviving fibers by sprouting of slow motoneurons (Delbono, 2003, 2011; Payne and Delbono, 2004; Aagaard et al., 2010; Reid et al., 2012; Venturelli et al., 2018). Interestingly, it is still debated whether the loss of motoneurons can be slowed down by regular physical activity [see Power et al. (2010) in favor and Piasecki et al. (2016) against this view]. Unfortunately, the direct assessment of the impact of neural events on muscle fiber size and number during advanced age and disuse is somewhat complicated (Doherty et al., 1993). However, the comparison between the force developed during maximal voluntary contraction (MVC) and electrically stimulated contraction helps to estimate the contribution of reduced neural drive to muscle deconditioning (Venturelli et al., 2015). Furthermore, the evaluation of *in vivo* single twitch kinetics may further contribute to understand the functional condition of skeletal muscle, as the maximal rates of force development are clearly different among slow and fast motor units (Mero et al., 1991). Unfortunately, information regarding single twitch kinetics in the oldest-old is sparse.

The authors apologize for this error and state that this does not change the scientific conclusions of the article in any way. The original article has been updated.
